# Abstract of the Proceedings of the Buffalo Medical Association

**Published:** 1858-12

**Authors:** Benj. F. Lemon


### ART. V.—*Abstract of Proceedings of the Buffalo Medical Association.*

TUESDAY EVENING, Nov. 2, 1858.

The Association met.

Present—The Vice-President, Dr. Newman, in the chair; Drs. White, Rochester, Gould, Wilcox, Rogers, Butler, Gay, Treat, Eastman, Miner, Lemon, Hutchins and Jeyte.

The secretary being absent, Dr. Lemon was appointed secretary *pro tem.*

The minutes of the last meeting were read and approved.

Dr. GOULD reported four cases of erysipelas treated by the use of tinct. ferri murias, applied externally as in the case reported by Prof. White at a former meeting of the association. He had previously been in the habit of prescribing the tinct. ferri internally, in doses of gtts. xv, every two hours, at the same time applying an ointment externally, composed of ferri sulph., ʒi, lard, ℥i, with satisfactory results in nearly every case.

CASE I. Miss —— had erysipelas of the face, extending over nearly the whole surface; the swelling and redness extending rapidly, and the vesicated portions very painful.

Applied the tr. ferri, externally, with a brush, three times a-day, and gtts. xv, every two hours, internally.

The pain and itching began to subside after the first application, and in a few days the patient reported herself well.

CASE II. Mrs. L—— had erysipelas of the face. It commenced on the right cheek, spread to the left ear, and covered half the forehead, before I saw it. The parts are much swollen, and the eyes nearly closed. Great pain and itching; tongue heavily coated; breath offensive; pulse over 100; and complained of great weakness and prostration.

This case looked badly, but I concluded to try the tr. ferri as in the last case, with essence of beef and wine.

In twenty-four hours there was evident improvement, and in a week the patient was discharged.

CASE III. P. F., an Irish laborer, aged 40 years, of intemperate habits, was attacked Sept. 24th, 1858. I first saw the case on the 27th. Face swollen, red, and large patches vesicated; pulse 100; thick brown coat on tongue; headache; anxious countenance; and loss of appetite. Expected delirium tremens as a complication.

Applied the tr. ferri every four hours, and gave gtts. xv, every two hours.

In twenty-four hours the patient began to convalesce, and in a week was discharged.

Case IV. B. M——, aged 26 years, was confined Oct. 9th, 1858. On the next day she was attacked with erysipelas on her left foot and leg, extending nearly to the knee. She complained much of pain and itching, and the limb was much swollen.

Applied tr. ferri every fours. Omitting it internally.

Two days after the first application there was less pain, itching and swelling; the skin looked shrivelled and dry. In a week the skin became dry and peeled off, leaving the whole surface smooth and healthy. In ten days from the commencement of the disease, the patient was discharged.

Very little additional treatment was needed in either of the four cases. The application appeared to control the local irritation at once, and convalescence followed in each case within two days.

Prof. Rochester related a case of "spina ventosa," in a child aged twenty months; it commenced in June last. An infant brother of the child had died of the same disease at the age of two years. He thought it probable that this one would not survive that age.

Dr. Lemon exhibited the "thyroid" and "cricoid" cartilages in a state of almost complete ossification. They were found in a subject about 45 years of age, in the dissecting room.

Prof. White had lately operated to close a "lacerated perinæum," using quilled sutures (pieces of bougie.) A catheter was left in the urethra, confined by adhesive plaster. The bowels were confined by opiates. In eleven days union was complete, and an alvine evacuation was procured by copious warm water enemata.

Prof. White had also lately removed a polypoid excrescence from the cavity of the uterus, by means of Recamier's instrument. The lady operated upon had long been troubled with watery evacuations from the womb.

After the removal of the excrescence, tincture of iron was applied. The watery discharges ceased from this time. The "os uteri" was easily dilated although the lady had uever borne children. The pain of the whole operation was inconsiderable.

Prof. ROCHESTER reported a case of inflammatory croup, which he had seen in consultation with Dr. Wilcox, in which the subject, a boy of seven years, recovered with difficulty. His mother lived in a basement, and the child had been subject to spasmodic croup. Last week, after playing all day, he was attacked after going to bed; his mother found him on his hands and knees and suffering from difficulty of breathing.

Drs. Rochester and Wilcox saw him at 9 o'clock, P. M. The respiration was hurried and difficult; cough hoarse and frequent; voice whispering, not husky; respirations about forty in the minute; great constitutional disturbance; no membranous exudation.

Made an application of the solution of the nitrate of silver to the fauces gave calomel and Dover's powder, and left an emetic of sulphate of zinc and ipecacuanha, to be given if necessary.

The next day the patient was found to have slight febrile movement, and six grains of Dover's powder were administered. The emetic had not been necessary.

He slowly recovered.

The parents, previous to asking advice, had given freely of "*Coxe's Hive Syrup*," from the effects of which he was much prostrated.

Prof. Rochester had seen cases in which he thought much injury had been done by the tartarized antimony: this case, he believed, would have resulted fatally if it had been continued.

Prof. WHITE was glad to see a decrease in the practice of giving antimony and other emetics in cases of croup, he thought it better, however, to err on the side of administering *mild* emetics than otherwise.

Prof. ROCHESTER preferred the zinc and ipecac. emetic, believing that it had not the prostrating effects of many others, and was more quick, sharp and mechanical in its action.

The Association then adjourned.

BENJ. F. LEMON, M. D.,
Sec'y *pro. tem.*

